# The Influence of Quality on eWOM: A Digital Transformation in Hotel Management

**DOI:** 10.3389/fpsyg.2020.612324

**Published:** 2021-01-14

**Authors:** Gloria Sánchez-González, Ana M. González-Fernández

**Affiliations:** Business Administration Department, University of León, León, Spain

**Keywords:** eWOM, objective quality, perceived quality, on-line comments, digital transformation, management, hotel sector

## Abstract

There is no doubt that the use of Internet for purchasing products and services has constituted a crucial change in how people go about buying them. In the era of digital transformation, the possibility of accessing information provided by other users about their personal experiences has taken on more weight in the selection and buying processes. On these lines, traditional word-of-mouth (WOM) has given way to electronic word-of-mouth (eWOM), which constitutes a major social change. This behavior is particularly relevant in the services area, where potential users cannot in advance assess what is on offer. There is an abundant literature analyzing the effects of eWOM on different variables of interest in this sector. However, little is known about the factors that determine eWOM. Thus, the main objective of the present paper is to analyze the impact of two variables (objective quality and perceived quality) on eWOM. Both of them are crucial for potential customers in the process of finding hotel accommodations and they can motivate people to make such comments. The results demonstrate that these variables truly have a significant impact on whether or not users make comments on line. Moreover, it proved possible to observe certain differences according to the profile of the tourist involved and the destination where the hotel is located. In the current changing environment, this information is of great use for hotel managers in order to design strategies according to the type of guest they wish to attract.

## Introduction

Over the last several decades, the Internet has consolidated itself as a world-wide basic means of communication, both for individuals and for businesses. Over time, the growing use of the Internet has implied that more and more customers are becoming hyper-connected across multiple channels, formats and device types, which in turn has meant an important digital transformation. The way people purchase products and services has changed, as they have access to abundant on-line shopping possibilities and a huge amount of information about the personal opinions of previous consumers. Through the Internet, people inform others about their personal experiences relating to certain products or services, information that the latter then use in taking decisions to make a purchase, whether on or off line.

This means of communication has become of particular value in the services area (Voyer and Ranaweera, 2015), with the hotel sector being among those most strongly affected (Serra-Cantallops and Salvi, 2014). The inseparability of the provision of a service and its consumption makes it particularly difficult to assess a service before making use of it (Litvin et al., 2008; Papathanassis and Knolle, 2011). Hence, having access to on-line messages significantly reduces uncertainty during the decision-making process in these circumstances (Yan et al., 2016; Hussain et al., 2020).

Taking advantage of this situation, a large number of tourist web pages allow their users to generate content in a simple, easy way, giving them control over the information itself, the manner in which it is distributed and the use that web surfers can make of it (Erdem and Cobanoglu, 2010; O’Connor, 2010). This brings with it easy access for potential customers to make comparisons of the quality of tourism services on offer.

As an outcome of this digital transformation, electronic word-of-mouth, termed eWOM, expands the possibilities for communication and the effects already present in traditional word-of-mouth during the purchasing process. Consequently, it has become an increasingly interesting field of study (Breazeale, 2009; Huete-Alcocer, 2017).

In the specific case of tourist accommodations, there are many pieces of work analyzing the effects of eWOM on different variables such as sales or profits (Gu et al., 2012; Yang et al., 2012a; Nguyen and Coudounaris, 2015), willingness to pay (Wu and Ayala-Gaytán, 2013), attitudes toward brands and products (Lee and Youn, 2009; Gavilán et al., 2018), or guests’ choice of accommodations (Noone and McGuire, 2014), among other topics. However, few studies have examined the factors that drive consumers’ eWOM behavior (Balasubramanian and Mahajan, 2001; Hennig-Thurau et al., 2004) and even fewer have done so with regard to the hotel sector (Hu and Kim, 2018; Yen and Tang, 2019), so there is ample space for more research.

Among the various determinants, service quality has proved to be an important factor in the marketing literature. Potential guests use these data during their decision-making process (Chan and Ngai, 2011), which justifies a more in-depth analysis of their effects on eWOM. Nonetheless, only two previous studies have analyzed its impact on eWOM activities in the hospitality industry (Yen and Tang, 2019; Serra-Cantallops et al., 2020) with no conclusive results. Hence, this paper has as its main aim to contribute to the pool of knowledge about the determinants of eWOM in this sector by investigating the influence of two kinds of quality: (a) objective quality and (b) perceived quality.

Previous research shows that the varied range of customers and hotels present makes it hard to reach generalizable conclusions (Chintagunta et al., 2010; Ghose et al., 2012; Gu et al., 2012; Blal and Sturman, 2014). Thus, these effects were additionally analyzed in relation to the profile of tourists. This has been demonstrated to be an important variable, for example, when modeling on-line review scores (Fang et al., 2016). In this case, travel companions were considered as the differentiating profile characteristic. Furthermore, as in Phillips et al. (2015), hotel location was also taken into account, with four European regions (Northern, Western, Southern and Central) being distinguished.

These features, as well as the use of a high-quality, reliable Europe-wide database, constitute a major contribution of the present paper in comparison to the vast majority of previous work which was in the form of location-based studies (Moro et al., 2017). All this favors generalization of the results and implies an advance in academic knowledge of eWOM. Likewise, from a managerial point of view, the results will be useful in designing strategies that improve the image of hotels and increase room bookings. Being constantly aware of factors that determine customers’ opinions and their preferences for hotel services can be the key to surviving in this digital era.

## Literature Review

As Internet became established and generalized, and e-commerce grew, there was a striking increase in its use when purchasing goods or services. This behavior has given rise to a new vehicle for exchanging information and opinions among consumers, so that traditional word-of-mouth (WOM), which has demonstrated to be more effective than other marketing techniques (Reichelt et al., 2014), has given way to electronic word-of-mouth (eWOM). It can be defined as “informal communication directed at consumers through Internet-based technology related to the usage or characteristics of particular goods and services, or their sellers. This includes communication between producers and consumers as well as those between consumers themselves” (Litvin et al., 2008, p. 461).

The specific nature of the Internet offers a huge variety of possibilities for communication, which has constituted a disruptive change relative to classic ways of buying (Cheung and Thadani, 2012; Yan et al., 2016). It allows access to information without limitations in time, as it can be synchronous or asynchronous (Litvin et al., 2008). Another characteristic relates to the number of individuals it connects: one-to-one (such as e-mails or instant messages), one-to-many (for example, websites), or many-to-many, as in the case of forums, blogs, virtual communities, and similar (Chan and Ngai, 2011; Moliner et al., 2015). Furthermore, this communication is no longer just among friends and acquaintances (Chan and Ngai, 2011), but can include contacts with numerous individuals, who may even be anonymous. On these lines, various marketing researchers have investigated the impact of such social ties in relation to consumers’ decision-making processes (Pasternak, 2017; Hussain et al., 2020). As a result of this communication, the way in which a decision to buy is made has changed considerably. Potential customers can access information about the features and uses of given products or services. Even more interesting, they can find out the opinions and assessments of people who have already bought and made use of them. Consequently, much more information is available to potential customers, which can be very useful to them in establishing their own perceptions of a business and its products (Li and Bernoff, 2008).

Specifically, in the tourism sector, this digital transformation has led to the sharing of opinions about personal experiences over the Internet as a widespread practice and eWOM has proven to be of great importance when the aim is to search for information about such experiences (Bronner and de Hoog, 2010). Since the early 2000s, a number of pieces of work concerning tourism services have highlighted the influence that recommendations from other consumers have over the making of a purchase decision by potential tourists (Litvin et al., 2005, 2008). In the particular case of hotel services, eWOM is a key aspect that requires great attention from managers, in order to carry out continuous improvements and develop a good reputation in the market (Park and Allen, 2013; Reyes-Menéndez et al., 2019). According to Bronner and de Hoog (2013), the importance of this type of information channel will increase when a product or service is characterized by three aspects: accessibility, relevance and experience, which is a perfectly fit in the case of hotel services.

From an academic perspective, studies have focused primarily on two dimensions of eWOM activities: eWOM volume and eWOM valence. On these lines, both the number of comments made by consumers and their ratings or feelings incorporated in their comments are key aspects. For example, Nieto et al. (2014, 2017) discovered that ratings and the number of reviews affected consumers’ decisions to purchase in tourism. They demonstrably improved profitability, satisfaction and business performance (Nieto et al., 2014) and affected the consumer’s willingness to pay for hotel services (Nieto et al., 2017).

With regard to the effects of the valence, there are no conclusive results so far. On the one hand, positive comments do improve attitudes toward hotels (Vermeulen and Seegers, 2009; Gavilán et al., 2018), and increase the number of reservations made (Torres et al., 2015), market share (Duverger, 2013), and sales (Duverger, 2013; Nguyen and Coudounaris, 2015). On the other hand, a hotel’s reputation becomes worse the greater the relative weight of negative comments, as against positive (Rose and Blodgett, 2016). Negative comments have a more persuasive effect than positive or neutral (Park and Nicolau, 2015). They seem more credible and impactful than positive views and this negative impact is stronger in the case of services than of physical goods. However, other authors conclude that complaints or negative comments are almost never used and the majority of the comments posted are recommendations (Bronner and de Hoog, 2010, 2013). Furthermore, a third possibility for eWOM can be considered, occurring when both positive and negative comments are present at the same time (Liang and Corkindale, 2019; Roy et al., 2019).

In respect of their number, prior research results seem to be clearer. It has been demonstrated that in general the volume of comments positively affects the sales of products, their popularity and awareness of them (Duan et al., 2008). In the case of hotels, the larger the total of on-line comments made about a hotel, the more positive views there are (Melián-González et al., 2013), the greater is preference for that hotel (Vermeulen and Seegers, 2009; Viglia et al., 2014) and the larger is the improvement in credibility (O’Connor, 2010). In consequence, the number of comments about hotels is an eWOM indicator that managers must keep strongly in mind. Its noteworthy influence on potential consumers’ opinions, especially in the case of products requiring information about previous experiences to ascertain their value (Yang et al., 2012a), merits a deeper understanding. For these reasons, this is the central variable of our study.

Research that addresses user-generated product reviews follows two major lines of investigation (Serra-Cantallops and Salvi, 2014): (a) the perspective of information senders, so as to analyze the motivations for generating and posting reviews (Cheung and Lee, 2012), and (b) the perspective of information receivers, so as to examine the adoption of such messages and the consequences for consumers and companies (Senecal and Nantel, 2004; Cheung et al., 2008). In marketing and communication literature, much has been written about the effects of eWOM. However, just a few studies have examined factors that drive consumers’ eWOM behavior (Balasubramanian and Mahajan, 2001; Hennig-Thurau et al., 2004). In this way, many authors have pointed out that despite its practical relevance, the antecedents of eWOM have received much less attention than its effects (Yang, 2013; Fu et al., 2015; Chu and Kim, 2018; Hussain et al., 2020). In the field of tourism, there are recent studies that go into the background of eWOM (Yang, 2013; Munar and Jacobsen, 2014; Lee and Oh, 2017; Dixit et al., 2019). Nevertheless, very few have done so specifically in the context of hotel services (Hu and Kim, 2018; Yen and Tang, 2019). Hence, the present paper attempts to address this research gap by focusing on the factors that lead to eWOM behavior in this sector.

The prior literature on motivations for eWOM examines the underlying personal determinants of individuals’ willingness to make such comments (*personal factors*). Regardless of the industry involved, the most important motives are entertainment, social ties, information, trust, social interaction, desire for economic incentives, interpersonal influence, concern for other consumers, and the potential usefulness of approval, among others (Hennig-Thurau et al., 2004; Chu and Kim, 2011; Lien and Cao, 2014). In the hospitality industry, factors such as altruism (Munar and Jacobsen, 2014; Bilgihan et al., 2016), enjoyment and economic incentives (Park et al., 2011; Dixit et al., 2019), sociodemographic characteristics (Nusair et al., 2011), social characteristics of the person (Kim and Tussyadiah, 2013), a sense of belonging to a community, social identity and a feeling of helping other consumers or enterprises (Serra-Cantallops and Salvi, 2014), have been identified.

However, little is known about the possible influence of other sorts of variables that might be called *specific service-related factors* (Serra-Cantallops et al., 2020). These determinants can differ, depending on the sector in question, because eWOM motivations are industry based (Harrison-Walker, 2001). According to Hofacker and Belanche (2016), it is important to analyze the antecedents and effects of eWOM, but it is also crucial to pay attention to those factors that may assist managerial efforts to encourage consumers to create content. Hence, in the hospitality sector, the characteristics of the hotel, which are specific service-related factors, may be more relevant than personal aspects from the point of view of hotel managers, because they control these factors (Serra-Cantallops et al., 2020).

In this way, one key aspect that should be considered is the quality of the service offered by the hotel (Serra-Cantallops et al., 2020). Service quality is a central marketing concept that has attracted continual research interest in the field of hospitality. It can be defined as “an overall judgment or an attitude relative to the superiority of a service” (Parasuraman et al., 1988, p. 16). On the one hand, many consumers resort to on-line comments to reduce risk and uncertainty when selecting a hotel and validating its quality (Kim et al., 2011). On the other hand, the quality of the service can also determine tourists’ opinions and their level of contentment. Parasuraman et al. (1985) believed that service quality is a result of comparisons between consumers’ expectations and the actual services provided. In accordance with the theory of expectancy disconfirmation, a comparison between prior expectations and the perceived level of service received during consumption constitutes the degree of satisfaction of customer (Parasuraman, 1997). In marketing literature, it is widely accepted that satisfaction and perceived quality are strongly interlinked and various previous studies demonstrate that quality has a significant impact on the degree of satisfaction and on consumer loyalty (Moliner et al., 2015; Ziqiong et al., 2015; Serra-Cantallops et al., 2018). In turn, these variables are related to an intention to make recommendations through inter-personal communication after a virtual purchase (Godes and Mayzlin, 2004; Moliner et al., 2015). Businesses fully understand this prime role for quality as a determining factor for making suggestions and comments on the Internet (eWOM). Firms in the tourist sector are putting great efforts into designing communication strategies for improving the quality of the services offered. However, academic research in this area is still far from extensive, as most of the studies have focused on restaurants and have hitherto yielded no conclusive results (Jeong and Jang, 2011; Zhang et al., 2014; Kim D. et al., 2015).

It would appear that only just two previous studies have analyzed the influence of service quality on eWOM behavior in the specific context of hotels. Yen and Tang (2019) deduced that a good performance of hotel’s attributes, as indicators of service quality, had a positive effect on eWOM. On the other hand, Serra-Cantallops et al. (2020), in the context of upper-class hotels, found no positive relationship between these two constructs. Their results showed that, although service quality is crucial for consumer satisfaction, it is not a determinant of positive eWOM. This lack of conclusive results makes the topic very interesting.

The present study builds on this literature stream. Following the recommendations of Serra-Cantallops et al. (2020) it is proposed to take a step forward in the analysis of the relationship between a hotel’s quality and eWOM by distinguishing between objective quality and perceived quality. In respect of the former, in the hotel sector the category or “star rating” assigned reflects this kind of quality, so that all potential tourists are likely to use it as an objective indicator for this feature. The existence of a standard classification of hotels gives potential customers an idea about the intrinsic quality of establishments, and allows managers to design different strategies based on the higher or lower category of their hotels (Öğüt and Tas, 2012). This variable is important from the eWOM viewpoint because there are differences in consumer behavior as an outcome of the objective quality assigned to the hotel. From the angle of eWOM adopters, previous studies have demonstrated that customers of top-category hotels (high objective quality) mostly select accommodations on the basis of strongly positive assessments, while for medium and low category hotels the overall number of on-line comments is more important (Blal and Sturman, 2014). However, little is known about the effects of this variable upon the decision to make online comments.

Various authors accept that European hotel classification constitute a measure of objective quality (Abrate et al., 2011). However, the process of standardizing categories for accommodations around the world is proving to be an arduous task. There are efforts to control and standardize the quality of hotels through a star rating. However, failures to review and update the category assigned to a given hotel make this assessment difficult. Thus, star ratings sometimes become an ambiguous signal of quality for tourists, which may cause differences between customers’ expectations and the star rating assigned to a hotel. For this reason, it is also crucial to take into account the quality perceived by customers.

It is possible to define perceived quality as the personal assessment made by a customer of the overall quality of the product or service received (Zeithaml, 1988). This is an important element determining consumer decisions (Susilowati and Sugandini, 2018), especially in the case of services. In these circumstances, the provider knows the real quality of the service while potential customers do not (Öğüt and Tas, 2012; Bronner and de Hoog, 2013), so they use this information as a benchmark for the hotel’s quality perceived by those who have already been guests (Bansal and Voyer, 2000). For this reason, on-line comments have become one of the most influential variables affecting brands (Rose and Blodgett, 2016). They come from customers who have previously made use of a service and voluntarily decide to express their opinions about it, which confers great credibility on their views (Sparks and Browning, 2011; Ye et al., 2011).

In this context, the attributes classically used as indicators of a hotel’s quality are cleanliness, location, services, characteristics of the room and the hotel in general, security, reputation, and the attentiveness of staff, among others (McCleary et al., 1993; Lockyer, 2005; Wilkins et al., 2010). All of these are important for tourist perceptions of quality, since they have an impact on brand value, their overall experience, their willingness to pay a given price, and on the process of building customer loyalty (Berry, 2000; O’Connor, 2010). One way of getting to know the quality perceived by guests relative to these attributes of a hotel is sentiment analysis. This methodology involves analyzing unstructured contents generated by users (Schuckert et al., 2015; Pelsmacker et al., 2018; Al-Natour and Turetken, 2020). It concentrates on a review of the text of comments made by tourists so as to identify the sense of their feelings toward the hotel involved (positive, neutral or negative), as also the intensity of these feelings (Kirilenko et al., 2018). From this analysis it is possible to identify their degree of satisfaction and the value they set on their experiences (Xiang et al., 2015; Berezina et al., 2016; Geetha et al., 2017). Similarly, it makes it feasible to learn their opinions on the specific characteristics of the hotel that give rise to that level of satisfaction or dissatisfaction (Sparks et al., 2016; Luo et al., 2020). This to some extent reflects the results of comparing their prior expectations about the hotel with their real experiences.

In academic circles there is growing interest in attempts to demonstrate a positive correlation between text comments and numerical ratings (Kim H.-S. et al., 2015; Lee et al., 2017; Yoon et al., 2019). This would allow unstructured qualitative data about users’ opinions to be transformed into quantitative scores. In the context of hotels, López Barbosa et al. (2015) used sentiment analysis to investigate the presence of correlations between numerical ratings and textual comments. The results they obtained confirmed there was such a relationship. In similar fashion, Geetha et al. (2017) examined the connections between the feelings expressed in text-based reviews and the number scores for two categories of hotels: premium and economy. Their results also showed that there was consistency between the scores given and clients’ actual feelings.

On this point, it is worth noting that on-line booking systems also allow access to the scores given and the comments made by former guests on the Internet about various aspects of hotels (Vermeulen and Seegers, 2009; Öğüt and Tas, 2012). It is therefore possible to make use of these scores as a good indicator of guests’ perceptions about a hotel’s quality when textual comments are not available, as is the present case. Ratings represent a guest’s level of satisfaction (Reichheld, 2003; Ganjalyan, 2018; Shen et al., 2018) and researchers in marketing agree that satisfaction and perceived quality are highly interconnected (Serra-Cantallops et al., 2020). These data can thus be used as indicators of this kind of quality. On similar lines, some previous studies have concluded that eWOM has a significant effect on perceived quality (Susilowati and Sugandini, 2018). However, there do not appear to be any previous studies considering the inverse relationship.

In view of the above, it seems clear that quality, whether objective or perceived, is a key element in the degree of satisfaction of tourists. Consequently, it may be surmised that it will have an impact on their intention to record their happiness or unhappiness with regard to these aspects through online comments. Hence, it is to be expected that both types of quality would influence the decision to take part in eWOM. On this assumption, the following may be hypothesized:

H1: The objective quality of a hotel has a significant influence on eWOM relating to it.

H2: The perceived quality of a hotel has a significant influence on eWOM relating to it.

**FIGURE 1:**
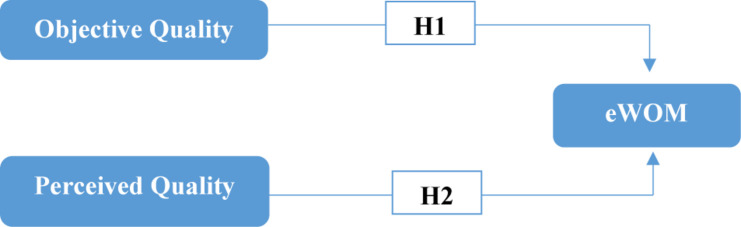
Hypothesized research model.

Figure 1 shows our research model:

As previously indicated, the expectations of hotel users may vary as a function of customer segment, hotel characteristics or both (Chintagunta et al., 2010; Gu et al., 2012; Blal and Sturman, 2014), so that is of some importance to take account of these aspects. Hence, analyses complementary to the two hypotheses quoted above were carried out. The aim was to investigate whether results differed as an outcome of the tourist profile (according to the travel companions) and a hotel’s geographical location within the European Union as North, West, South or Center, in accordance with the pattern of regions and sub-regions that the World Tourist Organization uses to divide up the map of world tourism (UNWTO, 2018).

## Data and Methodology

### Sample

There are currently a good number of on-line intermediaries that allow users to make hotel reservations directly in any part of the world. Among these websites, Booking.com was chosen for this study, as it is the world leader in on-line hotel reservations. It is a reliable, high-quality database, two important aspects when it comes to adopting information (Erkan and Evans, 2016). The site is available in forty-three languages and offers more than five million hotels, apartments and other types of accommodations, located in more than 120,000 destinations and 229 countries.

The website provides information both on an aggregate basis for all tourists having visited a given hotel, and by customers segments according to accompaniment. This latter distinguishes between people traveling alone, with friends, as a couple, or with children. Additionally, the site gives details of other variables, such as price, star rating and location of the hotel.

**FIGURE 2:**
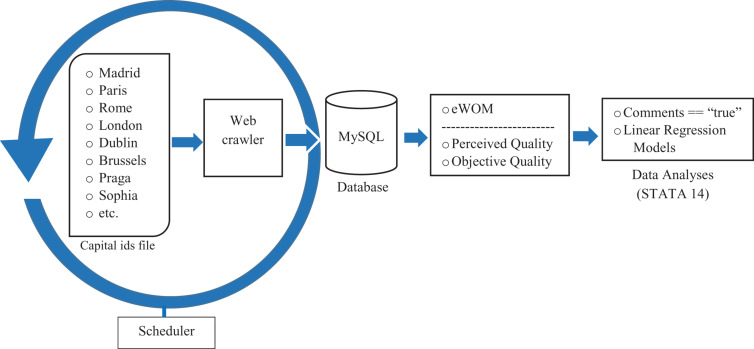
Data collection process.

Figure 2 shows schematically the process used to obtain data. The data collecting process took place in May 2018 and the services of a specialized company were hired to obtain the necessary data to carry out the present study. This company developed a software solution tailor-made for the project, a web-crawler^1^. This software made possible automatic extraction of the relevant details from each webpage and the creation of a database to organize the information gathered. The crawler was developed using *Ruby onRails* technology that receives the identity (*id*) of a capital city from Booking.com as a seed and simulates a search for it. The search results page directly yielded the necessary data. A MySQL database, created specifically for the task, stored all the information obtained.

The criteria indicated here were used to obtain the final sample. Since Booking.com permits reservations at hotels in any country in the world, the first criterion was to select hotels located in countries in the European Union. The next was to identify those situated in the capital cities of each of these countries^2^. Thereafter, STATA 14 was used to eliminate any hotels not providing data on all of the variables of interest. In this way, a final sample was compiled of 1,718,779 individuals who had posted a comment after a stay in a hotel reserved through Booking.com. The outcome was that in total 5,509 hotels were analyzed.

### Variables and Model

The aim was to determine the effects of different variables on eWOM. Consequently, in measuring the variables chosen, account was taken of the following criteria^3^. A discrete quantitative variable, the number of comments made for each of the hotels in European capital cities available through Booking.com, gave a value for the dependent variable, eWOM.

With regard to the explanatory variables, objective quality was measured by using a discrete quantitative variable reflecting the category of the hotel in terms of the number of stars (values from 1 to 5). This categorization is widely accepted in the European Union, with no major differences between the schemes for awarding stars used in the various countries (Arcarons i Simón et al., 2008). Secondly, it is widely accepted the use of online customer ratings as a signal of quality (Öğüt and Tas, 2012). Therefore, four indicators of perceived quality were utilized, these being: cleanliness, location, staff and the relationship between quality and price. These are continuous quantitative variables showing the scores given by customers on a scale from 0 to 10 (0 being the lowest score and 10 the highest).

In addition, the model included a series of control variables, one referring to price, four to the location of the hotel, and six relating to the profile of the customer. No previous studies analyzing the influence of these variables on eWOM appear to exist. Nevertheless, there is no doubt that they are key variables in the process of selecting a hotel, so that the model should include them. The price refers to the cost of a double room, and it is a continuous quantitative variable expressed in euros. With regard to the location of the hotel by sub-region of the European Union, four dummy variables were used (Central, Southern, Northern and Western), taking the value 1 when the hotel was located in the given sub-region and 0 when it was not. Finally, the model also included six variables representing the percentage of each type of customer relative to the total sample according to the profile of tourist^4^.

In the light of this information, the intention was to check the hypotheses put forward, using a linear regression model to determine the effects of the different variables on eWOM. Additionally, a second series of linear regression models is presented, taking into account the various segments of tourists and sub-regions of the European Union, with an eye to complementing the results obtained from the main analysis. All the statistical analyses were carried out using STATA 14.

## Results

The first linear regression model shown in Table 1 reflects the effects of the variables considered on the number of comments made (eWOM) for the sample of hotels in the European Union. This Table 1 includes both standardized Beta coefficients, and non-standardized coefficients, as well as Student’s *t*-values and levels of significance. In addition, robust standard errors were also calculated in order to control for heteroscedasticity in the model and ensure its robustness. In respect of the quality of the regression model, corrected *R*^2^ showed that the variables selected explained 21.6% of the variance of the dependent variable. The Snedecor F statistic had a value of 107.95. This demonstrates that there is a significant linear relationship between the dependent variable and the group of independent variables. Thus, the model proposed does serve to explain participation in eWOM by means of the variables chosen. Additionally, even though all variables were standardized, the variance inflation factors (VIFs) were checked in each of the regression equations. The data gave no evidence of multicollinearity, the highest Variance Inflation Factor (VIF) having a value of 4.62 (mean 4.55e + 12) for the pooled sample.

**TABLE 1:** Regression model for factors determining eWOM.

	Non-standardized coefficients		Beta standardized coefficients	Parametric tests	
			
	B	Robust standard errors		*t*	Sig.
Constant	−16.624	1.267	−	−13.26	0.000
Star rating	0.874	0.162	0.093	5.545	0.000
Cleanliness	−0.004	0.001	−0.072	−2.82	0.005
Location	0.007	0.001	0.152	11.41	0.000
Staff	−0.013	0.001	−0.227	−10.98	0.000
Quality-price	0.187	0.017	0.277	12.02	0.000
Price	−0.193	0.037	−0.108	−6.15	0.000
Southern	3.296	0.318	0.162	9.84	0.000
Northern	7.127	0.409	0.364	20.09	0.000
Western	6.616	0.359	0.366	18.98	0.000
Fam. small children	0.481	0.115	0.064	4.25	0.000
Fam. older children	1.410	0.120	0.177	11.81	0.000
Mature couples	1.038	0.102	0.145	9.79	0.000
Young couples	1.557	0.087	0.222	16.94	0.000
Groups of friends	1.734	0.255	0.101	7.20	0.000
	N	*R*^2^	*R*^2^_*a*_	*F*	Sig.
	5,509	0.216	0.214	107.95	0.000

**The variables “Central” and “Tourists traveling alone” acted as reference variables.**

**FIGURE 3:**
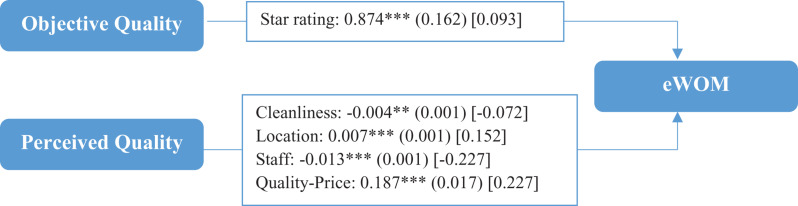
Regression model for factors determining eWOM. Results of the hypothesized model. ***p < 0.001; **p < 0.05. The values outside the brackets are unstandardized coefficients, those in parentheses are standard errors, and those in brackets are standardized coefficients.

The results obtained confirmed both of the hypotheses proposed in relation to objective quality and perceived quality, as shown in Figure 3. With regard to objective quality measured in terms of the star rating, a significant positive relationship was seen to exist between this quality and eWOM, bearing out hypothesis H1. This result indicates that the higher the objective quality of the hotel, the more comments customers make, which leads to the conclusion that tourists of this kind are more demanding. In respect of the second hypothesis, all the variables proved to be statistically significant, even if their signs varied, which would also confirm hypothesis H2. It is possible to see how some variables representing the quality perceived by tourists, such as scores for location and the price to quality relationship, had a significant positive influence. This implies that higher scores for these variables increased the number of comments, favoring eWOM. In contrast, higher ratings for cleanliness and staff had a significant negative impact on eWOM. In these cases, the higher the perceived quality in respect of these variables, the smaller the number of comments made by tourists. Thus, all of these variables are of importance for tourists when selecting hotels, since they have a significant influence on the number of comments, in some cases when the customer is satisfied and in others when the hotel fails to come up to expectations with regard to particular features.

The values for standardized Beta coefficients, which permit comparisons of the relative impact of the different variables included in the model independently of their measurement units, show that the variable representing the relationship between quality and price had the greatest influence on eWOM, followed by staff. The results indicate that customers were particularly interested in stressing their favorable opinions about the relationship between quality and price. In contrast, tourists made more comments if they were unhappy with the service or the treatment they received from the hotel’s staff.

With regard to the control variables, the first point of interest is that price has a significant negative influence on the number of comments. The higher the price, the fewer comments customers make. For its part, when the destination where the hotel was located lay in Northern or Western Europe, as against Central Europe, this had a significant positive influence over whether comments were made about it. Moreover, young couples were the most highly involved in eWOM activities, an unsurprising result because their age would make them more accustomed to using new technologies. The comments made in the following section go deeper into all these aspects.

In order to take into account the possible effects of tourist profile and to ensure if our main findings are robust, we estimate our model using several different subsamples according to travel companions. Table 2 shows the regression models taking into account the different profiles of travelers. In this case, the *R*^2^-values indicate that the variables selected explain 14.4, 17.1, 18.6, 11.2, 11.8, and 12.2% of the variance of the dependent variable for each type of tourist profile, respectively. In all the models Snedecor’s F statistic indicates the existence of a linear relationship between the dependent variable and the explanatory variables taken together. Hence, the six models proposed serve to explain the influence of the chosen variables over the number of comments (eWOM), according to the tourist profile.

**TABLE 2:** Regression model for factors determining eWOM by tourist profile.

	Families small children	Families older children	Mature couples	Young couples	Groups of friends	Tourists alone
Constant	−	−	−	−	−	−
Star rating	0.095***	0.056***	0.224***	0.121***	0.040*	0.094***
Cleanliness	−0.094***	−0.143***	−0.026	−0.102***	−0.150**	−0.061**
Location	0.116***	0.183***	0.198***	0.108***	0.158***	0.148***
Staff	−0.142***	−0.127***	−0.125***	−0.163***	−0.217***	−0.198***
Quality-price	0.383***	0.340***	0.249***	0.371***	0.295***	0.214***
Price	0.006	−0.015	−0.082***	−0.058**	−0.094***	−0.142***
Southern	0.194***	0.187***	0.239***	0.272***	0.131***	0.153***
Northern	0.289***	0.394***	0.385***	0.307***	0.330***	0.365***
Western	0.221***	0.242***	0.363***	0.334***	0.208***	0.412***
*N* = 5,509	*R*^2^	*R*^2^	*R*^2^	*R*^2^	*R*^2^	*R*^2^
R^2^	0.144	0.171	0.186	0.112	0.118	0.122
*F- Snedecor*	122.8***	148.4***	158.1***	86.4***	64.0***	83.7***

*** p < 0.001; ** p < 0.05: * p < 0.1. Beta standardized coefficients are presented. The models were calculated using robust standard errors.

These analyses also confirm the two proposed hypotheses, which guarantees the robustness of our results. As can be observed, the two explanatory variables have a significant effect on eWOM and their corresponding positive or negative effect remains the same as in the general model, independently of the tourist profile. By type of tourist, consideration of the standardized Beta coefficients indicates that, regardless of whether tourists were traveling alone or in company, the explanatory variable with greatest influence on the eWOM, and moreover with a positive impact, continued to be the relationship between quality and price.

However, it is possible to note some differences with regard to the second weightiest variable by tourist profile. For families with small children, young couples, groups of friends and people traveling alone, the second most important variable was staff, with a negative impact. Such guests made fewer comments the happier they were with the treatment they receive from hotel’s staff. In contrast, for families with older children, location became the second most influential variable, with a positive impact on the number of comments. The same was true for star ratings in the case of older couples, with a positive impact on eWOM, as these guests can be more demanding because of their personal status and the higher categories of the hotels they tend to use. However, it is striking that cleanliness was not a significant variable for this group of tourists. The explanation for this is likely that they were staying at hotels in the higher categories, where this aspect would be guaranteed.

With regard to the control variables, it should be noted that the absolute price was also not significant for families with children, whatever their ages, while the relationship between quality and price was of significance. With regard to location, it is possible to observe that tourists traveling to capital cities in Northern and Western Europe were those who made the largest number of comments. All categories of tourists shared this trend.

Finally, Table 3 shows the various models that take into account the sub-regions of Europe in which the hotels assessed were located. Because of the volume of information provided in this table, Table 4 offers a summary of the main results from these models to assist in interpreting them.

**TABLE 3:** Regression models for factors determining eWOM by tourist profile and hotel location.

	Central						Southern					
		
	SC	OC	MC	YC	GF	TA	SC	OC	MC	YC	GF	TA
Constant	−	−	−	−	−	−	−	−	−	−	−	−
Star rating	0.016	−0.022	0.219***	0.155***	0.027	0.109**	0.349***	0.263***	0.346***	0.277***	0.212***	0.308***
Cleanliness	−0.107**	−0.207***	−0.113**	−0.146***	−0.153***	0.026	−0.016	−0.083*	0.096**	0.007	−0.060	−0.010
Location	0.067*	0.212***	0.216***	0.093**	0.166***	0.157***	0.132***	0.235***	0.258***	0.144***	0.234***	0.163***
Staff	−0.090**	−0.053	−0.059	−0.090**	−0.203***	−0.169***	−0.170***	−0.122**	−0.242***	−0.225***	−0.289***	−0.251***
Quality-price	0.538***	0.459***	0.283***	0.359***	0.274***	0.142**	0.313***	0.324***	0.246***	0.365***	0.317***	0.298***
Price	0.105**	0.120**	0.074*	0.079**	0.094**	−0.029	−0.137***	−0.094**	−0.117**	−0.140***	−0.180***	−0.238***
N	1,267	1,267	1,267	1,267	1,267	1,267	1,261	1,261	1,261	1,261	1,261	1,261
*R*^2^	0.216	0.209	0.212	0.132	0.081	0.049	0.178	0.174	0.214	0.142	0.153	0.137

	**Western**						**Northern**					
		
	**SC**	**OC**	**MC**	**YC**	**GF**	**TA**	**SC**	**OC**	**MC**	**YC**	**GF**	**TA**

Constant	−	−	−	−	−	−	−	−	−	−	−	−
Star rating	0.019	0.0147	0.184***	0.006	−0.025	−0.015	−0.062*	−0.087**	0.091**	0.023	−0.102**	−0.022
Cleanliness	−0.132***	−0.160***	−0.065	−0.105**	−0.215***	−0.120**	−0.044	−0.067	−0.016	−0.123**	−0.101*	−0.28
Location	0.085**	0.143***	0.173***	0.067**	0.080**	0.133***	0.213***	0.213***	0.192***	0.172***	0.212***	0.211***
Staff	−0.100**	−0.097**	−0.066*	−0.133***	−0.128***	−0.200***	−0.250***	−0.284***	−0.136**	−0.251***	−0.283***	−0.216***
Quality-price	0.363***	0.357***	0.252***	0.354***	0.331***	0.235***	0.316***	0.249***	0.228***	0.366***	0.192***	0.116**
Price	0.111***	0.021	−0.040	0.046	−0.030	−0.082**	0.009	−0.016	−0.112*	−0.109**	−0.110**	−0.127***
N	1,856	1,856	1,856	1,856	1,856	1,856	1,125	1,125	1,125	1,125	1,125	1,125
*R*^2^	0.087	0.086	0.109	0.063	0.069	0.056	0.091	0.083	0.077	0.095	0.136	0.073

***p < 0.001; **p < 0.05; *p < 0.1. Beta standardized coefficients are presented. The models were calculated using robust standard errors. SC, Families with Small Children; OC, Families with Older Children; MC, Mature Couples; YC, Young Couples; GF, Groups of Friends; TA, Tourists traveling alone.

**TABLE 4:** Summary of the most influential variables by tourist profile and hotel location.

	Europe			
	
	Central	Southern	Western	Northern
Fam. small children	Quality-price (+)	Star rating (+)	Quality-price (+)	Quality-price (+)
Fam. older children	Quality-price (+)	Quality-price (+)	Quality-price (+)	Staff (−)
Mature couples	Quality-price (+)	Star rating (+)	Quality-price (+)	Quality-price (+)
Young couples	Quality-price (+)	Quality-price (+)	Quality-price (+)	Quality-price (+)
Groups of friends	Quality-price (+)	Quality-price (+)	Quality-price (+)	Staff (−)
Tourists traveling alone	Staff (−)	Star rating (+)	Quality-price (+)	Staff (−)

This is a preliminary analysis that will likely lead to future research. It is a first approach to studying the influence that may be exercised by profile characteristics of tourists and hotels over participation in eWOM. The results show that even if these market segmentations are incorporated, the two hypotheses put forward continue to be confirmed. In addition, the most striking result is that the relationship between quality and price was generally the variable with the greatest influence over the number of comments, having a positive effect, regardless of the sub-region in which the hotel evaluated was located and of the tourist profile. However, consideration of the second most influential variable for eWOM shows a greater number of differences (see Tables 3, 4).

In the case of Western Europe, the second most important variable for families with children, whatever their ages, and for groups of friends, was cleanliness, which exerts a negative effect on eWOM. Likewise, the staff variable had a negative influence on eWOM for young couples and people traveling alone. For mature couples, the objective quality (star rating) was the second most important variable, with a positive sign. In the case of Central Europe, the quality-price relation was also the most influential in all cases except for people traveling alone, for whom it was staff, with a negative impact. Additionally, there were clear differences by tourist profile in respect of the second most important variable.

In Southern and Northern Europe, the results were not so homogeneous, which prevents the drawing of general conclusions. In the first area, the South, quality-price relation and star rating came in the first two positions, varying between first and second by type of customer profile. For families with young children, older couples and people traveling alone, the variable star rating was the most influential. However, for all other groups, the relationship between quality and price held this position. In Northern Europe, the prime position went to the quality-price relation for families with small children, mature couples and young couples, with a positive effect here too. In the case of families with older children, groups of friends and people traveling alone, the first place fell to the variable staff, with a negative impact.

In view of the differences noted, it would be of great interest to investigate at some future point the effects on eWOM from other variables related to the profiles of tourists and characteristics of hotels. For example, it would be possible to consider any or all of the age, gender, educational level, and even country of origin of tourists, as also the age, number of rooms and location of hotels in countries in other continents. In this way, the door would be opened to undertake comparative analyses offering more precise and exhaustive results regarding the differences in preferences and behaviors of tourists.

## Discussion and Conclusion

The changing environment fostered by an increasing use of the Internet has implied huge numbers of changes in many aspects of people’s lives. One of the most visible is the way in which they buy products and services. Especially with regard to services, the Internet has made it possible to have access to information that would have been unimaginable just a few decades ago. On these lines, tourism is one of the sectors most affected by this situation. The main characteristics of tourism, the inseparability of provision from consumption and its intangibility, make it particularly difficult to assess such a service in advance (Grönroos, 2000). This brings with it a high perceived risk for potential customers during the decision-making process (Litvin et al., 2008). However, the use of the Internet and the communication possibilities that it offers is triggering noteworthy changes in the way in which people consume and plan journeys (Erdem and Cobanoglu, 2010; Papathanassis and Knolle, 2011). In this new era, electronic word-of-mouth (eWOM) has become a key element in the process of selecting and booking tourism services. Previous literature on this topic concentrated fundamentally on studying the effects that eWOM has on different variables of interest (Litvin et al., 2008; Ye et al., 2011; Öğüt and Tas, 2012; Melián-González et al., 2013; Viglia et al., 2014). Nonetheless, little research has investigated the factors influencing the making of on-line comments by customers.

Therefore, the present piece of work aims to contribute to existing knowledge in this field by studying the variables that promote participation by consumers in this sort of communication in the context of travel accommodations. In this sense, service quality has proved to be an important factor from the marketing point of view. However, just two previous studies have analyzed its influence as an eWOM determinant in the hospitality sector (Yen and Tang, 2019; Serra-Cantallops et al., 2020). Consequently, the present paper attempts to address this research gap by extending the analysis made by Serra-Cantallops et al. (2020) in that we consider the distinction between objective quality and perceived quality. These two variables are under the control of the hotel’s managers, which is crucial to develop more suitable marketing strategies according to the type of tourist that they are aimed to attract.

To accomplish this aim, the Booking.com website was used to create a European Union database corresponding to 5,509 hotels and covering all categories (star ratings). This in turn allows generalization of the results and constitutes a major new contribution to the literature published to date.

The study concentrated on analyzing a range of variables that can influence the number of comments made by customers as a function of their personal experience. Although the use of quantitative scores is an objective measure of on-line reviews, few previous studies have researched the scores when compared with textual comments (Moro et al., 2017). This aspect can also be considered a further contribution by the present paper.

Additionally, these effects were analyzed taking into account the profile of tourists (as a function of the people with whom they traveled), and of the geographical location of the hotels by European region (North, West, South, and Center). These two facts, in their turn, constitute a third contribution from this work, since previous studies have stressed that the great differences between hotels and types of tourist make it hard to achieve conclusive results (Chintagunta et al., 2010; Ghose et al., 2012; Gu et al., 2012; Blal and Sturman, 2014; Phillips et al., 2015; Fang et al., 2016).

The results obtained lead to three main ideas. The first is that both the objective and the perceived quality of the hotel have a significant influence on eWOM. The second is that it is possible to observe that customers are motivated to make comments more as an outcome of their own perceptions with regard to different aspects of the hotel (perceived quality) than on the basis of objective features. Finally, the third conclusion is that, regardless of the tourist profile and the location of the hotel, the quality-price relation variable generally has the greatest impact on the making of on-line comments, and its effect is positive in all cases.

With regard to the first conclusion, two issues arise. On the one hand, the significant positive effect exerted by objective quality on the number of comments leads to the conclusion that customers will be more or less demanding in accordance with the quality attributed to the hotel by its star rating. Thus, the higher the category of the hotel, the greater will be guests’ motivation to make comments about it. Thus, managers of top-category hotels should pay more attention to this subject, as their guests are more prone to make on-line comments about their hotel experience. On the other hand, when it comes to perceived quality, all the variables representing it have a significant impact on the number of comments. While better assessments of location and quality-price relation have a positive effect on eWOM, better assessments of cleanliness and staff have a negative impact, reducing eWOM. Thus, these are crucial variables for tourists when choosing hotels. No information is available about the sense of these comments because they are numerical scores, so it is not possible to make definitive claims in this respect. However, the logical expectation would be that higher ratings would lead to more positive comments, and lower ratings to more negative. Hence, an interpretation of these results might therefore be an assumption that high scores for location and the price-to-quality relationship not only would give rise to more comments but additionally that these would be favorable to the hotel. These results are in accord with those claiming that participation in eWOM grows when opinions are more extreme (Bansal and Voyer, 2000), in this instance when they are strongly positive. In contrast, cleanliness and staff exert a significant negative effect on the numbers of comments, which could mean that hotel guests would record on the Internet any deficiencies regarding these aspects.

Concerning the second conclusion, the previous statement is true whatever the tourist profile and the location of the hotel may be. This result is of especial value in the hotel context. It shows that subjective matters, such as perceptions about different features, motivate people to take part in eWOM more than do aspects of an objective nature, like star rating and price (information easily available to anyone interested in the hotel). Taking into account the difficulty of judging the quality of hotel service in advance, this study highlights the fact that this kind of information is what is of most interest to potential customers and what is crucial in their choice of a hotel.

The third conclusion is particularly true for young couples and tourists traveling as a family with children, whatever their ages, these results being in line with previous studies (Campo et al., 2010). However, two key aspects should be stressed. First, on the basis of tourist profile, there are certain differences in respect of other influential variables that can be interpreted better by considering the ages of the tourists, whether they are traveling with children or not, and what outlay per family unit is involved. For example, those traveling with older children are aware of the difficulties of getting around with this sort of family group. Therefore, it is not surprising that for this type of tourist the place where the hotel is sited turns out to be one of the features with the greatest influence over their opinion of a hotel and hence on the number of comments they make. A good location in relation to activities feasible with children in the destination chosen by this type of tourist seems to have a positive influence on the making of comments. However, whatever their ages may be, the absolute price is not significant for families with children. This leads to the conclusion that in such cases, the amount of money spent on accommodations is normally large because of the number of individuals in the family unit so, in these cases, the important feature is the quality-price relationship rather than the price in itself. Secondly, in the case of elderly couples, star rating becomes the second most influential variable, with a positive impact on eWOM. This type of customer may be expected to be particularly demanding, in view of their greater age and likely greater wealth. For this reason, the probability would be that the more a hotel matches up to their expectations as a function of its category, or indeed exceeds them, the more motivated they will be to record this fact in the form of on-line comments.

All these results can be of great use when hotels face intense competition due to a saturated market. Thus, a better understanding of the specific determining factors for eWOM will help hotel managers improve successful marketing strategies and enhance the attractiveness of their hotels (Chaochang et al., 2015; Serra-Cantallops et al., 2020). It is essential that management should constantly monitor and assess on-line customer reviews and scores, so as to identify what attributes generate customers’ positive or negative attitudes toward their hotel. Customers’ scores are a relatively simple and objective measure of guests’ opinions that can be easily accessed by potential customers as a first criterion for selecting a hotel. Thus, it would be advisable to pay special attention to low scores and negative comments, in order to improve or solve the deficiencies found by hotel guests. One choice for tackling this question might be to show that managers are concerned about customers’ opinions, for example, by trying to provide them with appropriate answers, or even more, by inviting customers to give additional information about their level of satisfaction with the hotel’s services (Del Chiappa et al., 2018). This might encourage customer loyalty, increasing hotel profits (Öğüt and Tas, 2012). The very fact of giving an answer shows an image of concern about the opinions of guests and enhances a hotel’s credibility (Rose and Blodgett, 2016). This point is crucial because credibility reduces perceived risk and the cost of information, increasing perceived quality.

Thus, in view of the information given above, the general recommendation to managers of any type of hotel would be to concentrate their efforts on improving features related to the quality perceived by customers and designing strategies differentiated by segments (for example, as a function of the stage in family life or the number of people traveling together as a group). This may aid in limiting the number of negative comments or scores and enhancing the number of positive ones by taking into account the type of variables most relevant for a given tourist profile. This recommendation is particularly important if comments or scores are negative (Guerreiro and Moro, 2017) and related to variables that are under management control (Rose and Blodgett, 2016), as is the present case. This is because positive or negative assessments made by customers about features controllable by a hotel’s managers have a considerable impact on reputation (Min et al., 2015) and thus on the possibilities of attracting future guests. An adequate response could mitigate negative effects. Even more, hotels do not necessarily lose customers who post a negative review. If they give a proper answer to negative comments from customers, perhaps through personal contact with them, they may turn them into satisfied future guests.

However, answering all customer reviews is time-consuming and costly, especially if they are negative reviews (Nguyen and Coudounaris, 2015). Thus, taking into account that not all users are equally important, the profile of the user could also be used as a discriminating factor. As Moro et al. (2017) suggest, it is crucial to frame the responses on the basis of user profiles and focus hotel managers’ attention on those specific users who are more likely to give lower scores. Making the effort to turn those negative scores into positive could lead to affirmative eWOM.

Another strategy might be to try to attract opinion leaders who would be favorable toward a hotel’s services, and encouraging them to post positive feedback (Yang et al., 2012b). Those recommendations could be of particular value for hotelsin the higher categories, since their customers are clearly more demanding. In the case of lower-range hotels, managers should pay extra attention to aspects relating to price, hygiene or cleanliness, and to living up to standards in relation to rooms (Brotherton, 2004). On these lines, previous research has demonstrated that for hotels in the lower categories, customers are more sensitive to the number of on-line comments, whilst for those in the higher categories the most influential point is the positive or negative valence of the comment (Blal and Sturman, 2014). Thus, in accordance with the kind of hotel, management actions for the first type of hotel (lower categories) should aim at getting more comments (logically, positive as far as possible). In the case of higher-category hotels, they should aim to increase satisfaction levels among customers. Because these guests make fewer comments, any they do make should be evidence for a high degree of satisfaction. This will yield a certain image of exclusivity, which is what customers are looking for in this sort of accommodations (Blal and Sturman, 2014).

Moreover, it is vital to be aware of different aspects of a hotel according to customer profiles. In view of the fact that in all instances these are variables controllable by the hotel management, a good match to requirements according to tourist type will result in more bookings. Additionally, a hotel’s location is also a point to take into account, as it may aid in identifying the most important aspects in accordance with the sort of tourists it is intended to attract.

Some limitations of this study have already been mentioned, but they should still be listed, alongside other points that may in themselves constitute new lines of research. It would be desirable to increase the number of explanatory variables for eWOM, so as to include the valence of the comments and to have more information about tourist profiles in order to provide more complete results and to be able to offer a more exhaustive interpretation of them. With regard to this last limitation, it would be advisable to have more data about the tourist profile relating to characteristics such as gender, age, level of studies, or even home country. This would allow interesting comparative analyses to be made by customer segment, the results of which would be very useful for hotel managements when designing more specific strategies on the basis of the sociodemographic profiles of the tourists they wish to attract. Likewise, it would also be advisable to expand the number of hotels considered, looking not only at the European Union, but also hotels located in other continents. A worldwide comparison might also yield interesting results on differences in tourist behavior. Similarly, it might also be useful to analyze the moderating effects of star rating and tourist profile on the results presented here. Finally, account should be taken of the fact that potential tourists may use different types of website, depending on the types of sub-decision they want to make, whether search-determined or experience-determined (Bronner and de Hoog, 2013). Moreover, they are also free to post their comments on domain-independent social media, like Twitter or Facebook. The present research is based on data derived from a domain-specific medium (Booking.com). Future lines of research could take into account, not only other sources of domain-specific media such as Hotels.com or Travelocity.com, but also information from domain-independent social media. This would make it possible to analyze whether the online channel might be an important variable in the degree of guest satisfaction.

## Data Availability Statement

The raw data supporting the conclusions of this article will be made available by the authors, without undue reservation.

## Author Contributions

GS-G contributed to the present manuscript in its conceptualization, methodology, formal analysis, investigation, writing – original draft, and funding acquisition. AG-F participated in its conceptualization, investigation, and writing – original draft. Both authors contributed to the article and approved the submitted version.

## Conflict of Interest

The authors declare that the research was conducted in the absence of any commercial or financial relationships that could be construed as a potential conflict of interest.

## Footnotes

1 A web-crawler is a system capable of running in a planned way through a set of resources (including videos, tables, and other items). These resources may be local or remote, for instance URLs. In general, the crawler downloads contents only if they appear relevant to its objective. Moreover, by definition, a web-crawler is able to discover resources that are related and relevant to its application.

2 These were Northern Europe (London, Dublin, Stockholm, Helsinki, and Copenhagen), Western Europe (Paris, Luxemburg, Brussels, Amsterdam, Berlin, and Vienna), Southern Europe (Madrid, Lisbon, Rome, Athens, Ljubljana, and Valletta) and Central Europe (Warsaw, Prague, Bratislava, Bucharest, Budapest, Sophia, Vilna, Riga, and Tallinn).

3 Certain variables were normalized using the following transformations: (1) the square root of the variables: number of comments, price, families with small children, families with older children, mature couples, young couples and tourists traveling alone, (2) the cube of: cleanliness, location and staff, (3) the square of: quality-price relation, and (4) the logarithm of groups of friends.

4 (a) Families with small children, (b) families with older children, (c) mature couples, (d) young couples, (e) groups of friends, and (f) tourists traveling alone.
